# How to Keep Well in Africa

**Published:** 1893-05-13

**Authors:** 


### How to Keep Well in Africa.

I.—PREVENTION.

Now that America is becoming populous if not crowded, and while Australia has ceased for the time to be adesirable colonizing ground, it is Africa that is the land of promise. So many temptations are offered to young men—sometimes, we must add, untruthfully and unscrupulously offered by "promoters" of companies, who know the difficulties and dangers of the land, but choose to dwell only upon its fertility and wealth—to seek their fortune in the Dark Continent, that it becomes important for all to know how best to retain that primary condition of success, good physical health. To this end we gladly welcome a little brown paper covered pamphlet by Mr. Horace Waller, whose experience of tropical Africa dates from the early sixties, which is published by Mr. Murray at the modest price of a shilling. The booklet has now entered upon its fifth edition, but considering how clear and practical it is, and how many people are now looking to Africa as their life's goal, we could wish that it had seen its fiftieth.

The advice given in it may be roughly indexed under two heads—common sense and quinine. We propose to deal at present with the former.

In the first place then, common sense must decide who ought to go to Africa at all. The common habit of sending ne'er-do-wells to countries which are in process of colonization is rather unjust to those countries, giving them a worse reputation both for health and morality than they deserve. Doubtless in every new venture Argonauts are needed, but the sooner they have done their work and disappeared the better. It is not Jason and his crew that lay firm and sure the foundations of new empires. As Mr. Waller says: "Any man of unsteady and vicious habits will save himself and all around him a great deal of trouble by carrying out the process of destruction in England, where he can terminate his existence in his own way. If he attempts to brazen it out in such districts as these which we treat upon, it is but an aggravated form of suicide." No more need be said on that subject. A constitution already shaken by self-indulgence is not worth the cost of shipping to Africa.

But there is another form of suicide which does not lend itself so readily to condemnation, yet it is no less reprehensible. There is a word to be said to every missionary: "Martyrdom may be a noble doom, but when churches and societies send you out to Africa it is not simply with the object of enabling you to become martyrs. You represent in your education and outfit a considerable sum of money, and it is only common business honesty to give some return for that money in civilising and christianizing the natives. That is what you most ardently desire to do, you say. Then take the proper means—means adapted to the fact that you are a European, neither brought up on native food nor acclimatized to the equatorial zone. Your living like an African may be intended to show your sense of brotherhood with the degraded heathen round you, but this brotherhood of the stomach is only a sort of boon-companionship reversed. Even the fasting and abstinence which you practised, as a matter of conscience, in England, should now, as a matter of conscience, be given up. Analyse the motives that tempt you to this self-denial, and see how much belongs to a humble and sincere desire to do God service and to benefit those around you; how much to the boast of the Pharisee, 'I fast thrice in the week.'" Dr. Livingstone, who had an iron constitution and a hardier training than almost any who have followed in his train, expressly protested against trying to live on native food. He himself made the experiment in one journey, and he never overcame the injurious effects of it. We remember hearing how once a party from the English Universities Mission visited the Church of Scotland mission station at Blantyre. Looking at the cultivated fields around, they said, not without a touch of scorn, "This is not a mission: it is a market garden." But what is the end? Blantyre has, indeed, its graveyard, its martyrs to duty; but on the whole those who dwell there enjoy good health, and the natives who are within reach of its influence learn many things beside the creed of Calvin. While at the other station these University missionaries, the flower of English culture, the poorest of whom was probably brought up in more luxury than any of the Scotchmen, tried to live in native huts on native food, and so died off like flies in frost. It is impossible, even if it were desirable, for Europeans to return to the condition of primitive man, and they often mar or end a useful career by making the attempt.

Of course it is, in the first place, well to be sure that one is sufficiently sound in wind and limb to dare the perils of the African climate. Some go there who are, under the most favourable conditions, foredoomed to death. Tendencies to epilepsy, insanity, nervous prostration, which would never have shown themselves at home, come out under the malarious influences of the tropics. It would be well if no man went to Central Africa without having undergone a searching examination in not only his own health history, but that of his family as well. Even temperament, which is a more deeply-rooted thing than many of us know, may seriously affect the chances of health. The active-minded man will live and escape disease where another, apparently as strongly built, will succumb. The *dolce far niente* is dangerous. When in camp it is better to botanise, sketch, shoot, do anything to keep mind and body on the alert than to lounge about, smoke, and "rest." Work is a tonic everywhere, but in Africa it is almost a specific anti-febrine.

Work, but in moderation. Constant, steady energy is one thing, feverish pushing is another. "Remember," says Mr. Waller, "that the tortoise goes ahead of the hare in Africa as a rule." Times will come when every nerve must be strained, therefore the tension should sometimes be relaxed. It is impossible for any writer thousands of miles away from the scene of trial to say specifically when to use the spur and when the curb; it is possible only to say, "take common-sense among your luggage, and if you can't get that in a convenient and portable form, then—stay at home."

Still there are hints which are sure to be of use. For one, do not, for the sake of doing without one or two porters, go without a properly-constructed tent, and if possible, a camp bedstead. Sleeping on the ground is bad economy. Wear a flannel sleeping suit, and do not too feverishly throw off blankets because it is hot when you go to bed. In the early morning the temperature falls suddenly, and then a chill may be caught, which means an attack of fever. Do not go out in the morning fasting. A cup of coffee will fortify the system, all the more if an egg is beaten up in it. Coffee is better than tea, the making of which requires a lady's hand; and we are speaking of places where ladies are not, and indeed, for their own health's sake, had better not be. Of course, wear flannel always next the skin, but add a broad linen waistband to repel the sun's rays, direct or reflected from the black alluvial soil, from the sensitive nerves of the abdominal region, and also protect the head from the chance of sunstroke. Do not let your interest in the future of Africa drive you to superintend too closely the breaking up of new ground. Rich as is the alluvial soil, plentiful as may be the crops it will one day bear, it means at present for Europeans, miasma in every spadeful. Do not let the Briton's instinctive love for his bath lead you to plunge into every lake or stream you see. The sudden chill to your heated frame may be repeated in a fit of ague before many hours are over. As, however, in spite of all precautions you may get wet and chilled, it is well to have among your luggage an india-rubber hot-water bottle, which can be filled with hot water when you camp, and placed at your back and feet. Always sleep under a mosquito curtain, even where there are no mosquitoes; it serves to keep out a deadlier foe—miasma. Finally, be cheerful; not rashly bold nor nervously timid; and if you feel unreasonably irritable, be sure that you are threatened with fever, and doctor yourself accordingly. We hope to give some hints as to this doctoring at a future time.

